# Proximity Effect induced transport Properties between MBE grown (Bi_1−x_Sb_x_)_2_Se_3_ Topological Insulators and Magnetic Insulator CoFe_2_O_4_

**DOI:** 10.1038/s41598-017-02662-8

**Published:** 2017-05-25

**Authors:** Shun-Yu Huang, Cheong-Wei Chong, Yi Tung, Tzu-Chin Chen, Ki-Chi Wu, Min-Kai Lee, Jung-Chun-Andrew Huang, Z. Li, H. Qiu

**Affiliations:** 1 Department of Physics, National Cheng Kung University, Tainan, 70101 Taiwan; 2 Instrument Development Center, National Cheng Kung University, Tainan, 70101 Taiwan; 3 Advanced Optoelectronic Technology Center (AOTC), National Cheng Kung University, Tainan, 70101 Taiwan; 4 Taiwan Consortium of Emergent Crystalline Materials, Ministry of Science and Technology, Taipei, 106 Taiwan; 5 School of Electronic Science and Applied Physics, HeFei University of Technology, Hefei, Anhui 230009 China

## Abstract

In this study, we investigate the proximity effect in topological insulator (TI) and magnetic insulator bilayer system. (Bi_1−x_Sb_x_)_2_Se_3_/CoFe_2_O_4_ (CFO) heterostructure was fabricated using molecular beam epitaxy and pulsed laser deposition system respectively. As revealed from the magnetoresistance measurement, the weak anti-localization (WAL) is strongly suppressed by proximity effect in (Bi_1−x_Sb_x_)_2_Se_3_/CFO interface. Modified Hikama-Larkin-Nagaoka equation was used to fit the WAL results so that the size of surface state gap can be extracted successfully. The temperature-dependent resistance of the heterostructures at small and large perpendicular magnetic fields were also measured and analyzed. The results indicate that the surface band gap can be induced in TI and continuously enlarged up to 9 T, indicating the gradual alignment of the magnetic moment in CFO under perpendicular magnetic field. The approaches and results accommodated in this work show that CFO can effectively magnetize (Bi_1−x_Sb_x_)_2_Se_3_ and the heterostructures are promising for TI-based spintronic device applications.

## Introduction

Topological insulators are new state of quantum matter that composed of a bulk band gap and a time reversal symmetry (TRS) protected gapless surface state^1–3^. Opening a band gap in the surface state or breaking the TRS are the key for exploring novel quantum phenomena and TI based devices. Generally speaking, magnetic ions doping and magnetic proximity effect are ways to open the gap of the surface state. The method of magnetic ion doping has been used intensively to introduce the ferromagnetic moments in TI and open the surface state gap, as reported by large literature^4–6^. Moreover, the gap opening can lead to new physical state, called quantum anomalous Hall effect^7–9^. However, magnetic ions could easily generate magnetic cluster, which leads to eliminating surface state. Another problem is low Curie temperature. To our knowledge, the Curie temperature of magnetic doped TI still below 100 K^9, 10^, which hardly to be used for practical device applications. On the other hand, magnetic proximity effect is a promising method to magnetize TI. Magnetic proximity effect is an extrinsic magnetization method^11–15^, which combines TI with a magnetic insulator (MI). The magnetic insulating layer provides magnetic moments and induces magnetization of TI layer. Compare with magnetic element doping, magnetic proximity effect provides numbers of benefits, including switching surface state gap, avoiding magnetic cluster, preserving TI intrinsic crystalline structure, etc^16, 17^. In addition, lots of literature pointed out that magnetic proximity effect could offer a higher magnetization temperature^18, 19^, that is beneficial for the TI-based spintronic application. Therefore, magnetic proximity effect may be a suitable method for magnetizing and opening surface gap of TI. However, the surface gap opening is hard to observe because of the proximity effect only exists in the interface between TI and magnetic insulator layers. For this reason, the angle-resolved photoemission spectroscopy is not an appropriate way to observe the proximity effect since only the electronic structure of top surface (a few nanometers thickness) can be measured.

Another method to observe the surface gap opening is magnetoresistance (MR) measurement. During the past few years, numerous literature have focused on MR transport and observed the suppression of weak anti-localization^11, 12^ However, those studies did not deliberate the gap size of surface state directly. To our knowledge, only limited studies discussed and used the modified Hikama-Larkin-Nagaoka (HLN)^20–23^ to obtain the gap size. Therefore, in proximity effect of TI/MI, it’s a challenge to research on the gap opening in the TI surface state. Meanwhile, in low temperature range, both the quantum interference (contributed by the WAL and the weak localization (WL)) and electron-electron interaction strongly influence the temperature-dependent conductivity under external magnetic fields (σ(T, B)). In other words, analysis on σ(T, B) may also provide information regarding the surface gap opening, when massive Dirac fermions are considered. However, to our knowledge, there is still no experimental work clearly identifies this issue about the relationship between the slope change of σ(T, B) and the induced surface gap.

In this paper, we report the transport properties of (Bi_1−x_Sb_x_)_2_Se_3_/CoFe_2_O_4_ heterostructure, which fabricated using molecular beam epitaxy and pulsed laser deposition system, respectively. CoFe_2_O_4_ (CFO) has high Curie temperature and is an ideal insulating material that suitable to magnetize TI layer and use for MR measurement of the bilayer structure. Bi_2_Se_3_ is a prototype three-dimensional topological insulator and has been the subject of extensive research. However, most Bi_2_Se_3_ films suffer a serious problem, which has high n-type bulk carrier concentration due to the presence of Se vacancies that act as donors. Sb doping into Bi_2_Se_3_ can effectively reduce the vacancies of selenium and anti-site of bismuth and selenium. For this reason, Sb doping can greatly reduce carrier concentration of Bi_2_Se_3_^24, 25^ and here we choose Sb-doped Bi_2_Se_3_ as TI layer. After all, we analyzed and fitted the MR using modified HLN equation. The results show that CFO can magnetize TI layer and open the gap of TI. The phase coherence length *l*_*ϕ*_ of TI also decreases, which indicate that the electron of surface state strongly scattered by magnetic moment. Moreover, we measured and fitted the temperature dependence of conductivity at different magnetic fields in low temperature regime. The results indicate that the slopes κ reveal two different behaviors in small and large perpendicular magnetic field. In the small magnetic field, the κ will sharply increase with increasing magnetic field, which consistent with literature. However, in large magnetic fields, the κ decreases with increasing magnetic field. These results are significantly different with single layer results, which can be attributed to the continual enhancement of the surface gap size in the large magnetic field. Our findings should be useful for the future studies of magnetic TI and TI-based spintronic devices.

## Results and Discussion

**Figure 1:**
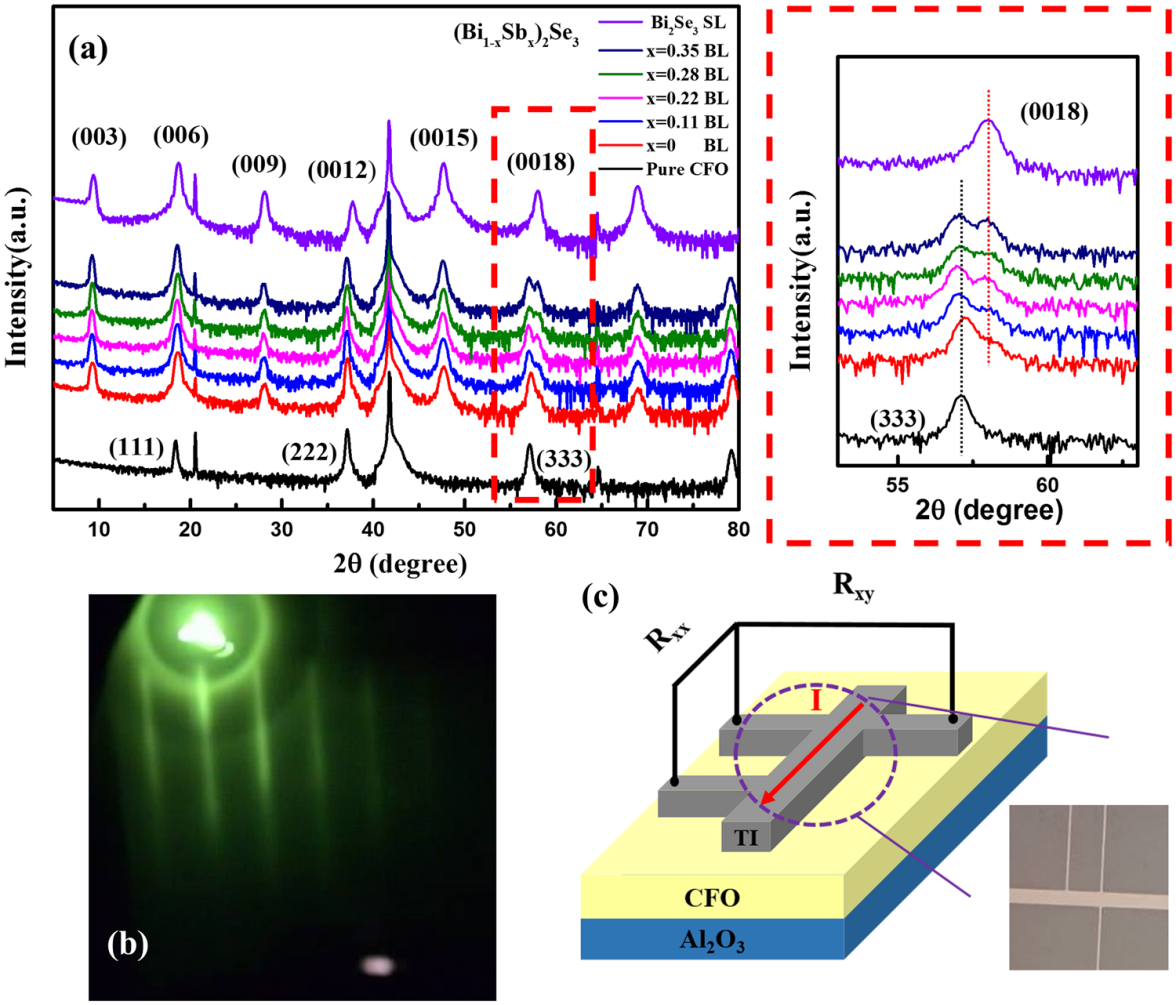
(**a**) X-ray diffraction patterns of single layer Bi_2_Se_3_, CoFe_2_O_4_ and bilayer (Bi_1-x_Sb_x_)_2_Se_3_/CFO samples. (**b**) The RHEED patterns of Bi_2_Se_3_ which grew on CFO layer. (**c**) The schematic diagram of bilayer structure, and the inset is Hall bar pattern which observed by optic microscope.

For the structural analysis, Fig. 1(a) shows the X-ray diffraction of the single layer Bi_2_Se_3_, CoFe_2_O_4_, and bilayer (Bi_1−x_Sb_x_)_2_Se_3_/CFO samples. All peaks can be identified with (00n) diffraction peak of (Bi_1−x_Sb_x_)_2_Se_3_, while CFO shows the (111) series diffraction peak. No other phases were observed in the heterostructure samples, which indicates that Sb was perfectly doped into Bi_2_Se_3_ in all the samples. Meanwhile, magnification of the diffraction peaks around 57.5° (show on the right inside the dashed rectangle) also reveal that the ideal interface and layer structure can be formed in (Bi_1−x_Sb_x_)_2_Se_3_/CFO heterostructures. Figure 1(b) shows the RHEED pattern of (Bi_1−x_Sb_x_)_2_Se_3_ layer, further confirming the high crystalline quality of the (Bi_1−x_Sb_x_)_2_Se_3_/CFO heterostructures samples. The structural analysis reveals that all (Bi_1−x_Sb_x_)_2_Se_3_/CFO heterostructures are ideal for electrical transport measurement.

**Figure 2:**
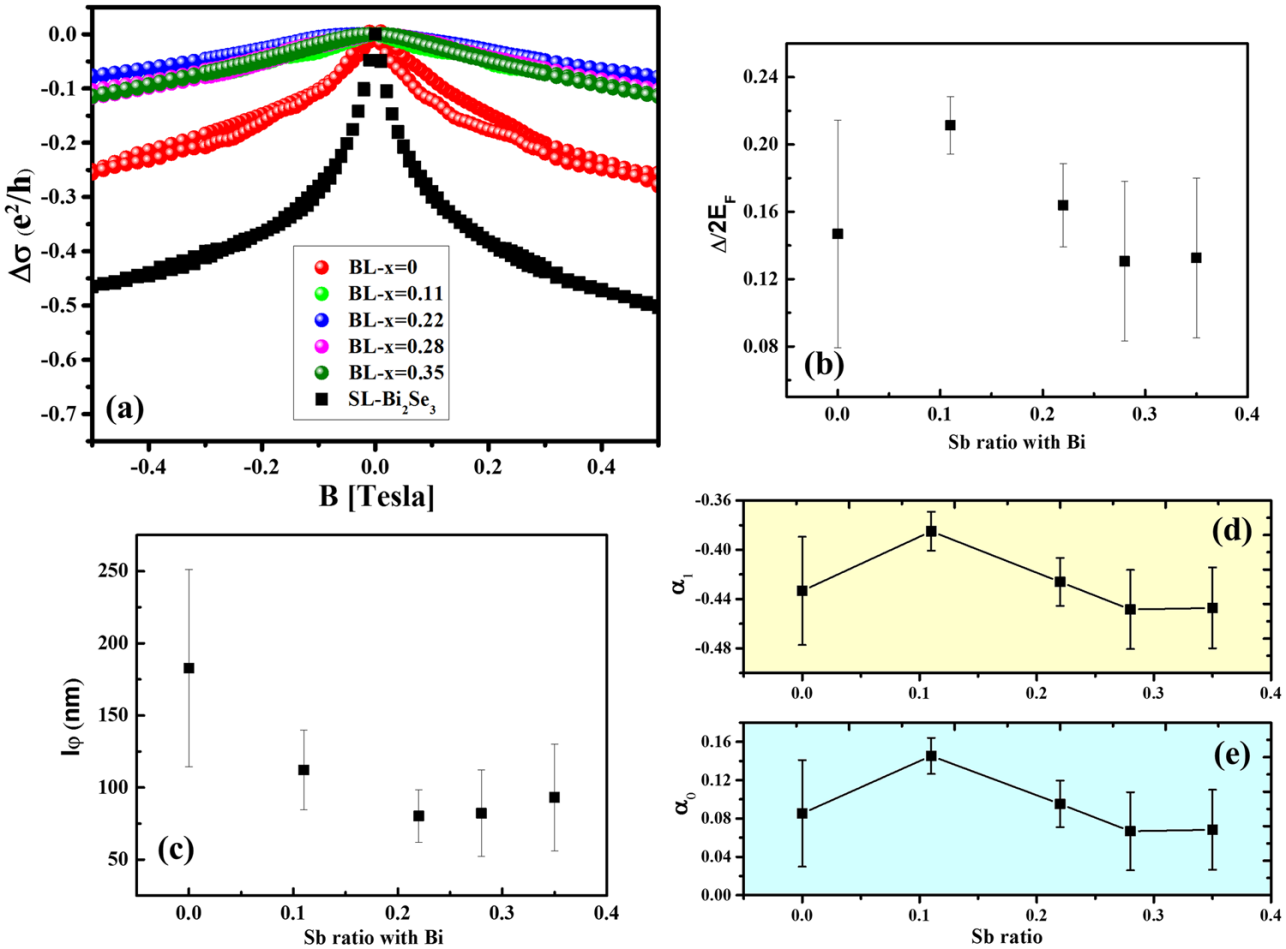
Magnetoresistance measurements and fitting results of the magnetoconductivity in perpendicular magnetic fields. (**a**) Normalized MC of the single layer (Bi_2_Se_3_) sample (squares) and bilayer (Bi_2_Se_3_:Sb/CFO) samples (circles) at 2 K in the low field region. (**b**) The fitted Δ/2*E*_*F*_ with different Sb doping. (**c**) The fitted phase coherence length with different Sb doping. (**d**) and (**e**) display the fitted *α*_1_ (upper panel) and *α*_0_ (lower panel) as a function Sb ratio.

Next we focus on transport properties of (Bi_1−x_Sb_x_)_2_Se_3_/CoFe_2_O_4_ heterostructures. Figure 1(c) schematically illustrates the device configuration of (Bi_1−x_Sb_x_)_2_Se_3_/CoFe_2_O_4_ heterostructures for MR measurement. The Hall bar was patterned using photolithography, which is observed by optical microscope as shown inside the dashed circle. Figure 2(a) presents the normalized magnetoconductance (MC) of the single layer (Bi_2_Se_3_) sample (squares) and bilayer ((Bi_1−x_Sb_x_)_2_Se_3_/CFO) sample (circles) at 2 K in the low magnetic field region. With perpendicular magnetic field, the MC of Bi_2_Se_3_ single layer exhibits weak anti-localization (WAL) behavior with a sharp cusp at low field region. In contrast to single layer Bi_2_Se_3_, the bilayer samples illustrate that the MC cusp feature disappears completely which means WAL was suppressed by being placed in proximity with the CFO. One of the origin for the suppression of WAL in TI is the enhancement of sheet resistance R_s_, where disorder could drive the crossover from quantum diffusive into strong localization regime^26, 27^. Here we estimate the disorder level by taking dimensionless conductivity, g ≡ σ/(e^2^/h) where σ = 1/R_s_. Even at intermediate disorder regime (1<g<3), deviation of α from the ordinary value (≥|0.5|) could be observed^26, 27^. However, our transport data reveals a g ≥16 (see Supplementary Figure S1c), which fulfills the condition for quantum diffusive transport (where g>>1). We thus attribute the observed MC to the quantum interference effect^28^. To understand the origin of weakened WAL cusp in bilayer samples, we carry out a quantitative analysis of low-field MC data. The modified HLN equation^22, 23^ has been manipulated to fit the MC data,

1 $${\rm{\Delta }}{\rm{\sigma }}({\rm{B}})=\sigma (B)-\sigma (0)=\sum _{i=0,1}\frac{{\alpha }_{i}{e}^{2}}{\pi h}[\Psi (\frac{1}{2}+\frac{{l}_{B}^{2}}{{l}_{\varphi i}^{2}})-\,\mathrm{ln}(\frac{{l}_{B}^{2}}{{l}_{\varphi i}^{2}})]$$

where Ψ is digamma function, the magnetic length ${l}_{B}^{2}=\hslash /4e|B|$, $1/{l}_{\varphi i}^{2}=1/{l}_{\varphi }^{2}+1/{l}_{i}^{2}$ and *l*_*ϕ*_ is phase coherence length. The modified HLN equation has two terms, in general, one is for weak localization (WL, i = 0) and the other is for weak anti-localization (WAL, i = 1), which contributes two groups parameter *α*_0_, *l*_0_ and *α*_1_, *l*_1_. In the WAL limit, *α*_0_ = 0, α_1_ = −0.5 while in the WL limit, *α*_0_ = 0.5, α_1_ = 0. *α*_0_, *α*_1_, *l*_0_, *l*_1_ are all related to cos *θ* = Δ/2*E*_*F*_, where Δ is the gap size of the surface state and *E*_*F*_ is the Fermi level. Figure 2(b) shows the fitting result of Δ/2*E*_*F*_ as a function of the Sb ratio for all (Bi_1−x_Sb_x_)_2_Se_3_/CoFe_2_O_4_ heterostructures and the fitting range was chosen between ±0.5 T. First, we can easily observe that all the extracted Δ/2*E*_*F*_ are above zero, which means that (Bi_1−x_Sb_x_)_2_Se_3_ layer has been magnetized by CoFe_2_O_4_ and a surface gap was induced in (Bi_1−x_Sb_x_)_2_Se_3_. To estimate the surface gap size, we use (Bi_0.65_Sb_0.35_)_2_Se_3_/CFO for the calculation since the surface E_F_ (n_2D_ ∼ 7.8 × 10^12^ cm^−2^) was below the conduction band edge (Fig. S1). By taking 2D Fermi wave vector of k_F_ = (2πn_2D_)^1/2^, we estimate E_F_^29, 30^ and calculate the surface gap where ${\rm{\Delta }}=2{E}_{F}/0.132\approx 51\,meV.$ Interestingly, the Δ/2*E*_*F*_ increases at the beginning, but decreases with continuously increasing the Sb ratio. As shown in the Hall measurement (Fig. S1), the E_F_ was actually shifted down in Bi_2_Se_3_ due to the Sb doping (with reduced carrier concentration). Therefore, the reduction of Δ/2*E*_*F*_ implies that the Δ should become smaller with increasing the Sb doping level.

To further elucidate the Sb doping effect to the MR behavior, the fitted phase coherence length *l*_*ϕ*_ vs. Sb ratio is examined. As shown in Figure 2(c), *l*_*ϕ*_ reduces with increasing the Sb ratio, but the tendency has been slowed down at high Sb ratios. The decreasing of *l*_*ϕ*_ may indicate strong magnetic scattering occurred at the interface of (Bi_1−x_Sb_x_)_2_Se_3_/CoFe_2_O_4_ that enhanced as a result of Sb doping. This observation suggests that the magnetic proximity effect has been enhanced in these heterostructures. Nevertheless, the Δ shows completely different trends as described above. This sounds counter-intuitive, since Sb is a non-magnetic dopant that is not expected to have influence on Δ. Here we propose a scenario as follow: in 3D TI layer, not only the bottom but also top surface state (SS) can contribute in MR behaviors. Owing to the proximity effect, the bottom SS is magnetized, leading to the TRS breaking induced surface gap, as indicated in the WL behavior. However, the top SS remains gapless, presenting spin-momentum locked (SML) massless Dirac fermions immune to backscattering (that resulting in WAL). Therefore, we deduce that the two transport mechanisms at different surface states are competitive. In our previous Sb doped Bi_2_Se_3_ studies, we have observed the phenomena where WAL increases with increasing Sb doping ratio due to the reduction of carrier concentration^31^. We thus suggest the slight decrease of the Δ with further increasing Sb doping ratio was attributed to the enhanced dominance of the WAL (top SS) over WL behavior (bottom SS). This finding indicates that there might be an interplay between the SML spin carriers and the magnetized interfacial spins. We summarize the extracted *α*_0_ and *α*_1_ as a function of Sb doping ratio as shown in Fig. 2(d,e) respectively. In our results, we can observe *α*_1_ ∼ −0.44 and *α*_0_ ∼ 0.08, revealing again that TI has been magnetized by CFO because *α*_1_ and *α*_0_ are more than −0.5 and 0, respectively. These results indicate that the proximity effect can slightly suppress WAL, which is consistent with the above-proposed scenario. Therefore, we deduce that the induced magnetic moments of (Bi_1−x_Sb_x_)_2_Se_3_ may not contribute in the perpendicular direction. The dependence of Δ/2*E*_*F*_ on Sb doping ratio further implies CFO may induce more in-plane magnetic moments in TI layer when increasing Sb content.

Nevertheless, the fitting range of modified HLN equation is strongly limited by ${B}_{\phi }=\,\hslash /(4e{l}_{\phi }^{2})$ which is the critical magnetic field characterized by the phase coherent length *l*_*ϕ*_. Therefore, the modified HLN equation can only describe low magnetic field range. Following, we employ the temperature-dependent resistance of the heterostructures at small and large perpendicular magnetic fields to further investigate the proximity effect^32–34^.

**Figure 3:**
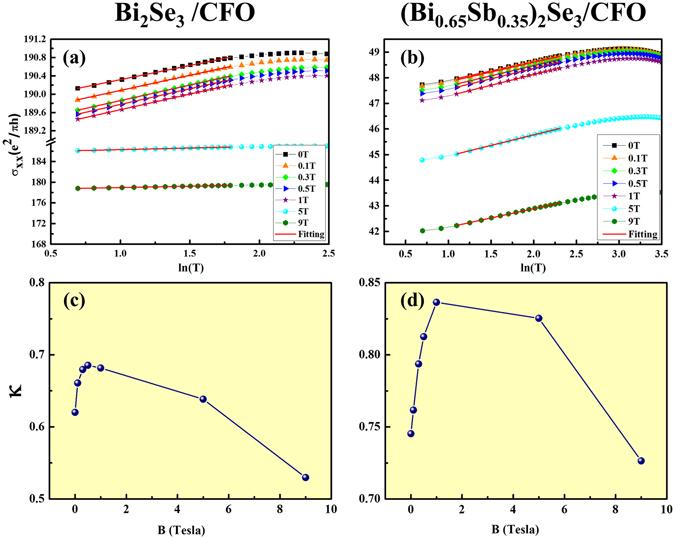
Temperature-dependent *σ*_*xx*_ of (**a**) Bi_2_Se_3_/CFO and (**b**) Bi_2_Se_3_Sb(0.6)/CFO samples are evaluated at B = 0, 0.1, 0.3, 0.5, 1, 5, and 9 T. The slopes are defined as ${\rm{\kappa }}=(\pi h/{e}^{2}){\rm{\Delta }}{\sigma }_{xx}(B,T)/d(lnT)$, which is plotted as a function of B in (**c**) Bi_2_Se_3_/CFO and (**d**) Bi_2_Se_3_Sb(0.6)/CFO sample.

Figure 3(a,b) show the temperature-dependent conductivity of Bi_2_Se_3_/CFO and (Bi_0.65_Sb_0.35_)_2_Se_3_/CFO respectively, where normalized conductivity vs. lnT at different perpendicular magnetic fields (from 0 T to 9 T) are plotted. We fitted the low temperature range data to get slope κ, which defines as κ = (*πh*/*e* ^2^)∂*σ*/∂*lnT* and plotted as a function of B shown in Fig. 3(c,d) for Bi_2_Se_3_/CFO and (Bi_0.65_Sb_0.35_)_2_Se_3_/CFO, respectively. Figure 3(c,d) shows the similar tendency, where κ at first increases in low magnetic fields, but decreases with further increasing the magnetic fields. Lu *et al*.^32^ had pointed out that κ are dominated by two different effects. One is the electron-electron interaction (EEI) and the other is quantum interference (QI) effect. In low field range, both QI and EEI contribute to κ. However, QI can be destroyed by further increasing perpendicular magnetic field and leads to increasing of κ. In our result, κ of Bi_2_Se_3_/CFO and (Bi_0.65_Sb_0.35_)_2_Se_3_/CFO are saturated in 0.5 T and 1 T respectively, which is determined by the magnitude of *l*_*ϕ*_. In high field range, only the EEI contribute to κ and the slope of EEI can be described as κ_*EEI*_ = 1−*η*_Λ_ *F*, where *η*_Λ_ = (1 + cos^2^ *θ*)/2 and *F* is electron screening factor. In general, the κ_*EEI*_ should be constant in magnetic fields. However, in our data, the κ decreases at high magnetic fields, which means that the *η*_Λ_ and the *F* must be influenced by magnetic fields. The theoretical studies indicate that *η*_Λ_ and *F* are functions of the surface gap^32^. Therefore, the decreasing of κ at high magnetic fields is due to the proximity-induced surface gap, revealing that the CFO has induced more magnetic moments in TI layer at high magnetic field. Considering the magnetic anisotropy of CFO, here we propose a mechanism for the high field enhanced proximity effect, as explained in the following section.

**Figure 4:**
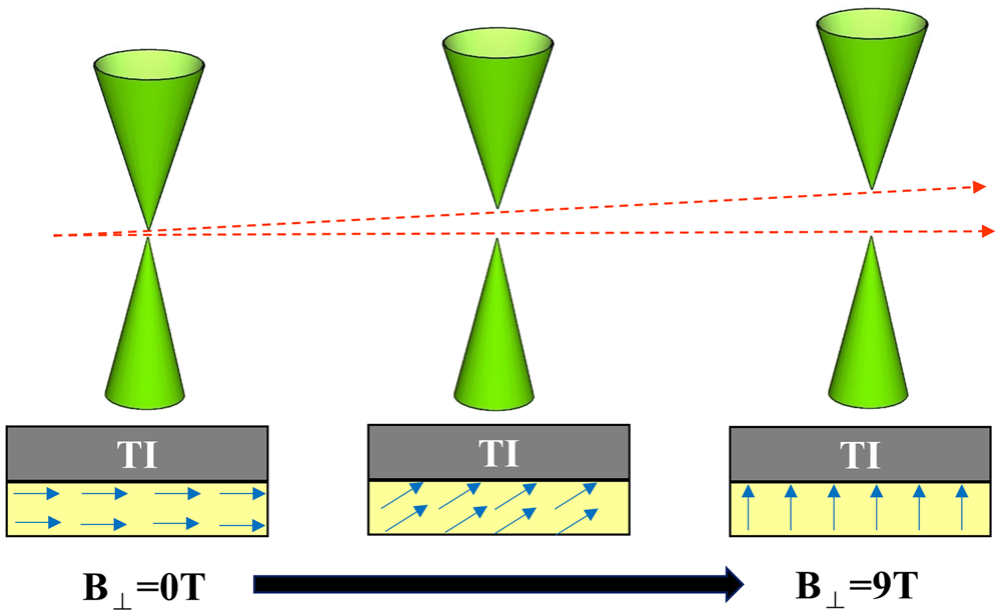
Schematic diagram of the bilayer structures in perpendicular external magnetic fields. The diagram shows that the gap size of surface state can increase with increasing the external B fields.

Figure 4 schematically illustrates the relationship between surface gap size and magnitude of perpendicular magnetic fields of (Bi_1−x_Sb_x_)_2_Se_3_/CoFe_2_O_4_ heterostructures. Because the CFO easy axis is at in-plane direction (Figure S3), the magnetic moments are more likely to lie in plane at small external perpendicular magnetic fields. Consequently, only weak magnetization of topological insulator and smaller band gap is induced. While the perpendicular external magnetic field further increases, the magnetic moments of CFO align to the external B field gradually. Due to the larger perpendicular component of magnetic moments, the magnetization of topological insulator would be stronger and the surface gap opening would be extensive. Therefore, we can observe the phenomenon of surface band gap opening through analysis of temperature-dependent conductivity at different magnetic field.

Controlling the TI surface state gap^35^ is a prominent process for spintronic and related devices^36–38^. For instance, by varying the surface gap using perpendicular magnetic fields, spin texture and the spin polarization of TI’s surface state can be effectively tuned. This tunability can be utilized in spintronic devices because the spin polarization rate is the key for spin transport. In addition, heterojunctions of magnetic-doped TI and magnetic insulator are promising candidates for proximity-induced high-temperature magnetic order in TI channel. Our results and methodology of fabricating the TI/MI heterostructures provide not only a platform for investigating the surface gap opening in TI, but a versatile method to explore the practical applications based on magnetic topological insulator.

## Conclusion

In conclusion, we successfully grew (Bi_1−x_Sb_x_)_2_Se_3_/CFO heterostructures on the c-plane sapphire substrate and the crystal orientation is well defined by XRD. In the bilayer system, the proximity effect can be observed clearly via magnetoresistance measurements. The gap size per E_F_ of surface state can be obtained using modified HLN equation fitting for all bilayer samples, where the surface gap is estimated about 51 meV for (Bi_0.65_Sb_0.35_)_2_Se_3_/CFO. In large perpendicular magnetic fields, analysis on the temperature-dependent conductivity reveals that the gap size can be enlarged by increasing external B field, which is caused by the magnetic moments of CFO rotating from in-plane to out-of-plane. This work offers a method to control the gap size of TI surface state in (Bi_1−x_Sb_x_)_2_Se_3_/CFO heterostructures by magnetic proximity effect.

## Materials and Methods

The CFO films were grown on c-plane sapphire in a 1.5 × 10^−5^ torr O_2_ ambient at 750 °C by pulse laser deposition (PLD) system and the thickness of CFO films were around 50 nm. The CFO film were then *in situ* transferred from PLD to molecular beam epitaxy (MBE) system in UHV condition. 15 QL-thick topological insulator (Bi_1−x_Sb_x_)_2_Se_3_ layers were grown on the CFO layer at 290 °C with base pressure of ∼1 × 10^−10^ torr. Surface morphology was monitored *in-situ* by reflection high energy electron diffraction (RHEED). After growth, the samples were capped with 2 nm Se for protecting the TI layer in MBE chamber. We varied the Sb doping ratio of (Bi_1−x_Sb_x_)_2_Se_3_/CFO samples with five different Sb fractions, namely x = 0, 0.11, 0.22, 0.28, 0.35. The crystal structure of all samples was confirmed by X-ray diffraction (XRD). To investigate the magnetic response and electric properties of (Bi_1−x_Sb_x_)_2_Se_3_/CFO bilayer structure, the samples were patterned into standard Hall bar devices with 100 μm length and 50 μm width by photolithography. The Hall effect measurements at 2 K indicate that the carrier concentration of the Bi_2_Se_3_/CFO and (Bi_0.65_Sb_0.35_)_2_Se_3_/CFO samples were ∼2.97 × 10^13^ cm^−2^ and 7.8 × 10^12^ cm^−2^ respectively (Figure S1). Four-probe magneto-transport and temperature-dependent resistance measurements were conducted using Physical Properties Measurement System (PPMS).

## Electronic supplementary material

Supporting Information
