# Supplementary material for: Proximity Effect induced transport Properties between MBE grown (Bi1−xSbx)2Se3 Topological Insulators and Magnetic Insulator CoFe2O4
Source: Sci Rep. 2017 May 25;7:2422. doi: 10.1038/s41598-017-02662-8 (PMC5445069; doi:10.1038/s41598-017-02662-8)
Supplement: Supplementary file 1 — Supporting Information [file 41598_2017_2662_MOESM1_ESM.pdf]

Proximity Effect induced transport Properties between MBE grown $(Bi_{1-x}Sb_x)_2Se_3$ Topological Insulators and Magnetic Insulator $CoFe_2O_4$

Shun-Yu Huang, Cheong-Wei Chong, Yi Tung, Tzu-Chin Chen, Ki-Chi Wu, Min-Kai Lee, Jung-Chun-Andrew Huang, Z. Li, H. Qiu

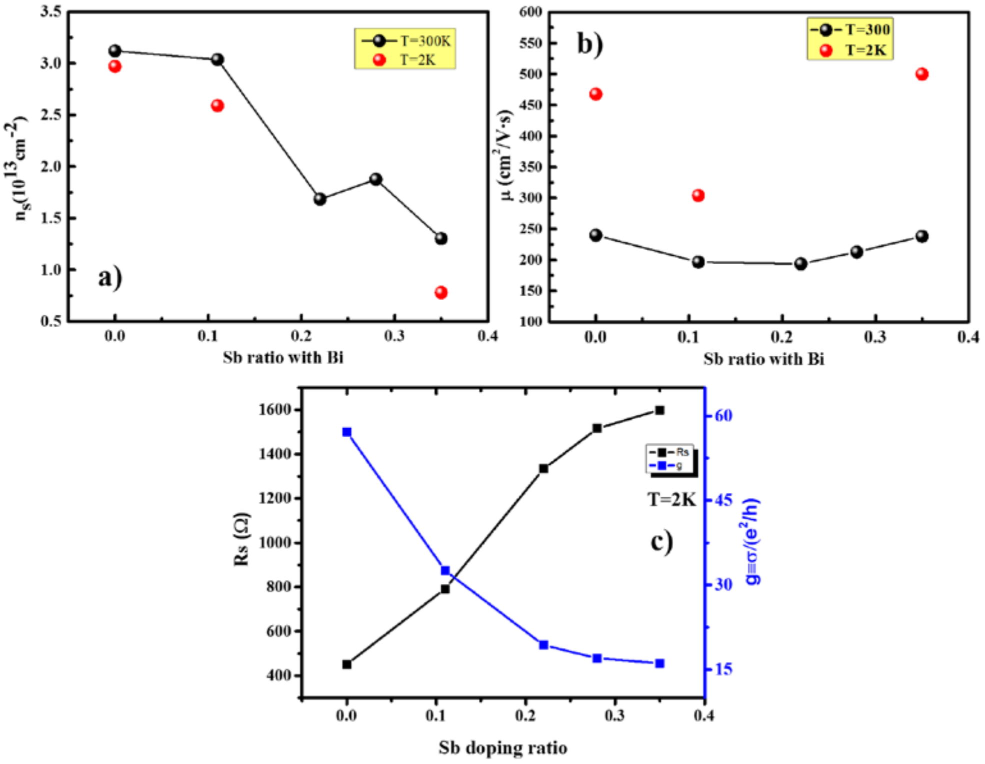

Figure S1. (a) 2D carrier concentration (n_{2D}) and (b) mobility μ at 300K and 2K; (c) sheet resistance R_s and dimensionless conductivity g of $(Bi_{1-x}Sb_x)_2Se_3$ in heterostructures (at 2K).

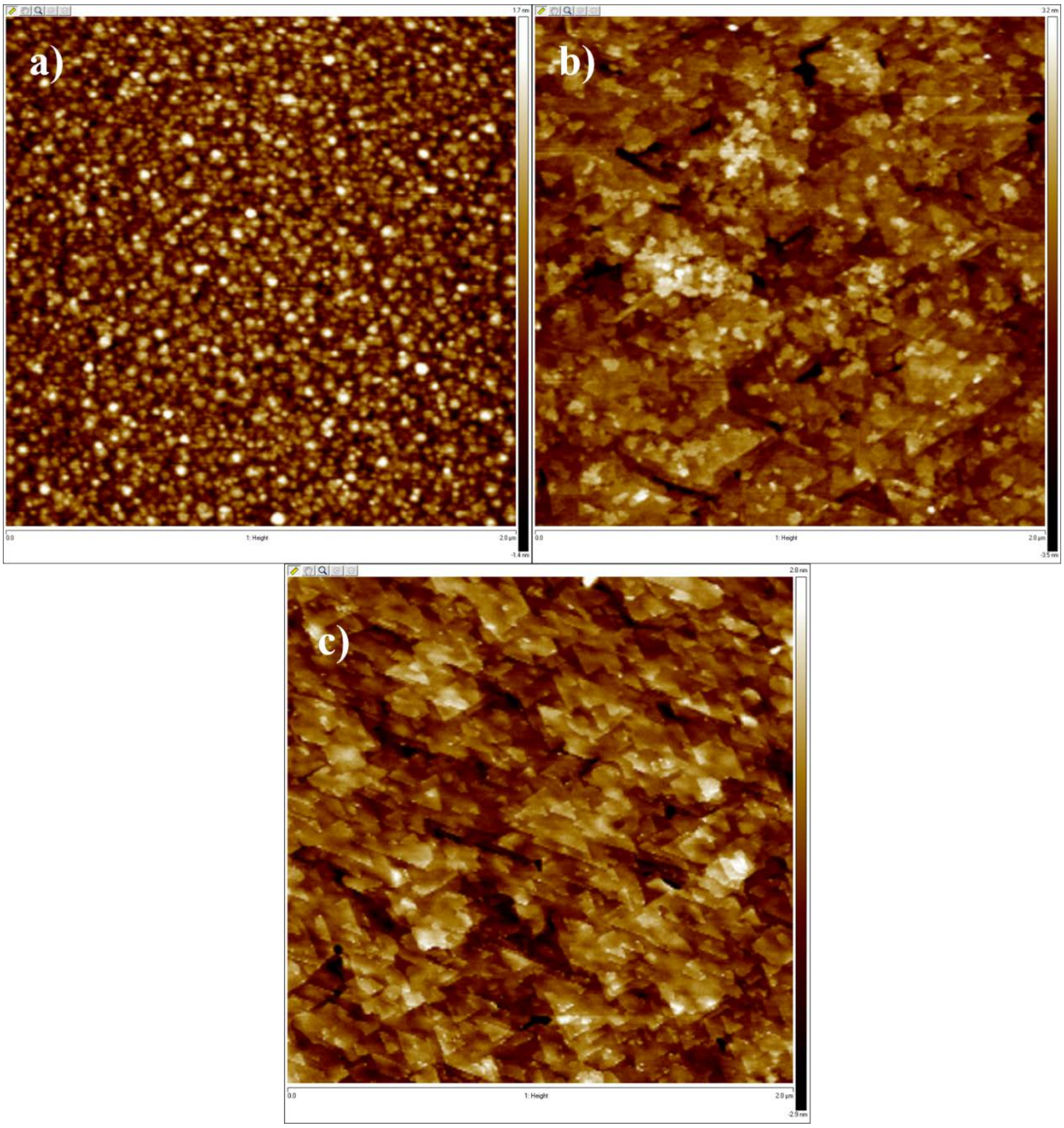

Figure S2. AFM image of (a) single layer CFO, (b) Bi_2Se_3/CFO bilayer and (c) $(Bi_{0.78}Sb_{0.22})_2Se_3$/CFO bilayer. The roughness of (a), (b) and (c) are about 0.53nm, 0.87nm and 1.07nm, respectively.

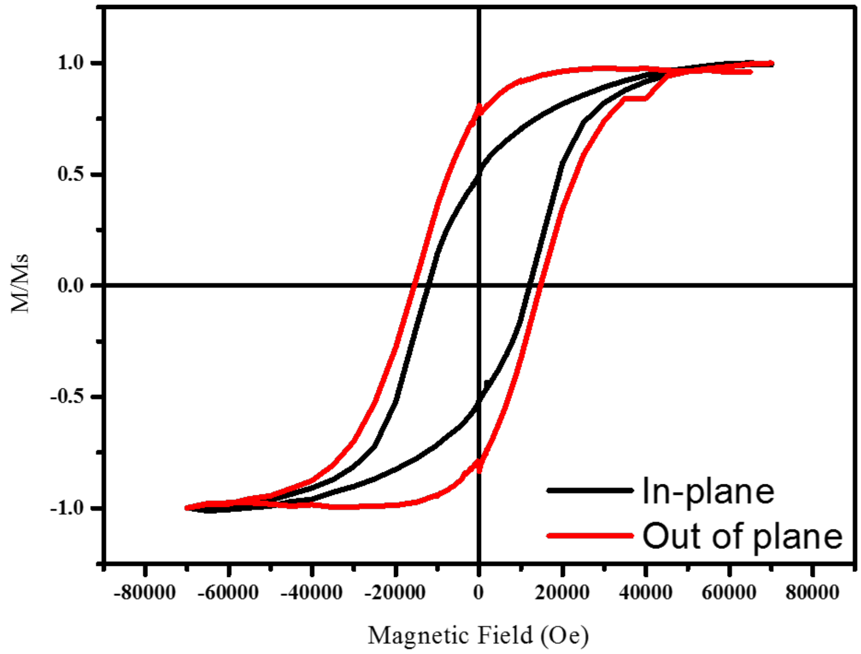

Figure S3. The M-H loops of CFO single layer with in-plane and out of plane direction at 2K.
